# Replication-inducible vaccinia virus vectors with enhanced safety *in vivo*

**DOI:** 10.1371/journal.pone.0230711

**Published:** 2020-04-02

**Authors:** Caitlin M. O’Connell, Brittany Jasperse, Caitlin J. Hagen, Allison Titong, Paulo H. Verardi

**Affiliations:** Department of Pathobiology and Veterinary Science and Center of Excellence for Vaccine Research, College of Agriculture, Health and Natural Resources, University of Connecticut, Storrs, Connecticut, United States of America; CDC, UNITED STATES

## Abstract

Vaccinia virus (VACV) has been used extensively as the vaccine against smallpox and as a viral vector for the development of recombinant vaccines and cancer therapies. Replication-competent, non-attenuated VACVs induce strong, long-lived humoral and cell-mediated immune responses and can be effective oncolytic vectors. However, complications from uncontrolled VACV replication in vaccinees and their close contacts can be severe, particularly in individuals with predisposing conditions. In an effort to develop replication-competent VACV vectors with improved safety, we placed VACV late genes encoding core or virion morphogenesis proteins under the control of *tet* operon elements to regulate their expression with tetracycline antibiotics. These replication-inducible VACVs would only express the selected genes in the presence of tetracyclines. VACVs inducibly expressing the A3L or A6L genes replicated indistinguishably from wild-type VACV in the presence of tetracyclines, whereas there was no evidence of replication in the absence of antibiotics. These outcomes were reflected in mice, where the VACV inducibly expressing the A6L gene caused weight loss and mortality equivalent to wild-type VACV in the presence of tetracyclines. In the absence of tetracyclines, mice were protected from weight loss and mortality, and viral replication was not detected. These findings indicate that replication-inducible VACVs based on the conditional expression of the A3L or A6L genes can be used for the development of safer, next-generation live VACV vectors and vaccines. The design allows for administration of replication-inducible VACV in the absence of tetracyclines (as a replication-defective vector) or in the presence of tetracyclines (as a replication-competent vector) with enhanced safety.

## Introduction

*Vaccinia virus* (VACV) is the prototype member of the genus *Orthopoxvirus* within the family *Poxviridae*, which also includes *Cowpox virus*, *Monkeypox virus*, and most notably *Variola virus*, the causative agent of smallpox. Immunization with VACV led to the successful eradication of smallpox worldwide [1] and since then, VACV has been used as a viral vector for the development of recombinant vaccines for humans and animals, cancer immunotherapies, and oncolytic therapies. Moreover, there is still significant interest in the development of next-generation smallpox vaccines to be used in case of a bioterrorist event or the emergence of other orthopoxvirus threats such as monkeypox [2–4]. However, the safety of live VACV vectors is always a concern, as severe complications such as accidental infection of the eye, eczema vaccinatum, progressive vaccinia, and post-vaccinial encephalitis [5–9] can result from uncontrolled virus replication after vaccination or therapeutic use. The occurrence of complications correlates with pre-existing conditions such as atopic dermatitis, cardiac disease, and immunosuppression due to infection (such as HIV/AIDS) or drug therapy. Consequently, individuals that have such conditions or those with contacts that have these conditions are contraindicated for vaccination or treatment with replication-competent VACVs [9–11].

A number of strategies have enhanced the safety of VACV vectors. These include the selection of natural strains with lower virulence, the development of highly-attenuated strains such as modified vaccinia Ankara (MVA) and NYVAC [12, 13], inactivation of virulence factors such as the thymidine kinase gene [14], deletion of immunomodulatory and other non-essential viral genes [15, 16], and expression of attenuating genes such as cytokines [17–20]. However, these safety improvements can lead to a reduction in the effectiveness of the vectors, as vaccine efficacy is typically compromised with attenuation [21]. In addition, next-generation smallpox vaccines must provide immunogenicity in clinical trials equivalent to licensed (replication-competent) vaccines, the effectiveness of oncolytic VACV vectors is contingent upon the replication competence of the vector, and overexpression of cytokines can lead to unforeseen immune activation and complications [22, 23].

In a continuing effort to improve VACV vectors, a new approach is to develop replication-inducible VACV vectors that are significantly safer yet replicate to the same levels as their parental strains, and therefore maintain their full immunogenic and oncolytic potential. These replication-inducible VACV vectors are based on elements of the transposon Tn*10* operon that confers tetracycline resistance in bacteria. Tetracyclines are broad-spectrum antibiotics that inhibit translation in gram-positive, gram-negative, and atypical bacteria [24]. In the *tet* operon, the Tet repressor (TetR) is unable to bind to *tet* operators in the presence of tetracyclines, allowing transcription of the tetracycline resistance gene [25]. The *tet* operon has been adapted to a variety of organisms for inducible gene expression, including prokaryotic, yeast, insect, plant, and mammalian cells, as well as transgenic organisms and viruses [26–32]. Control of gene expression in VACV has been achieved by expressing the TetR gene (*tetR*) constitutively and inserting a *tet* operator element (O_2_) after the transcriptional start site of VACV genes, allowing their expression to be regulated by tetracyclines [33, 34]. Therefore, we propose to improve the safety of VACV *in vivo* by using *tetR* and *tet*O_2_ to control the transcription of genes that are required for viral replication. Ideally, these replication-inducible VACVs should replicate to wild-type levels in the presence of tetracyclines such as doxycycline (DOX) and would be unable to replicate in the absence of these antibiotics.

Our previous work on tetracycline-dependent expression of VACV early transcription factors [35] led us to explore the control of other genes essential to VACV replication. In this study, three VACV genes (E8R, A3L, and A6L) conserved among all chordopoxviruses [36] that encode virion core proteins or a protein involved in virion morphogenesis were selected as candidate genes to generate replication-inducible VACVs. The specific role of the VACV E8R gene has not yet been accurately determined. E8 was first investigated as a potential membrane protein involved in endoplasmic reticulum (ER) wrapping [37]. A subsequent study indicated that E8 is made early in infection and might mediate the binding of DNA to ER membranes [38], while a later study reported that E8 is expressed late in infection and has a potential role in early transcription within the viral core [39]. The A3L gene encodes the precursor to protein 4b, a major virion component that localizes to the inner core wall [40–43]. A3 has a role in virion morphogenesis, particularly in the transition from immature particles to intracellular mature virions [44]. A6 is a viral membrane assembly protein [45] and a minor virion component [46, 47] expressed late in infection and appears to be essential in virion morphogenesis, specifically crescent formation, through its lipid binding function [48–51]. A3, A6, and E8 are core-associated proteins present both in intracellular mature virions (MVs) and extracellular enveloped virions (EVs).

We used elements of the *tet* operon to design and construct VACVs that inducibly express the E8R, A3L, or A6L genes. We evaluated and characterized the properties of these VACVs and showed that viruses inducibly expressing A3L, A6L, or E8R replicate indistinguishably from wild-type VACV in the presence of tetracyclines, and more importantly, that viruses inducibly expressing A3L or A6L only abortively infect cells in the absence of antibiotics. Furthermore, a VACV inducibly expressing A6L was not detected in mice infected in the absence of tetracyclines but caused weight loss and mortality similar to wild-type VACV in the presence of tetracyclines.

## Results

### Design of *tet*-responsive late promoters and construction of recombinant viruses

**Fig 1:**
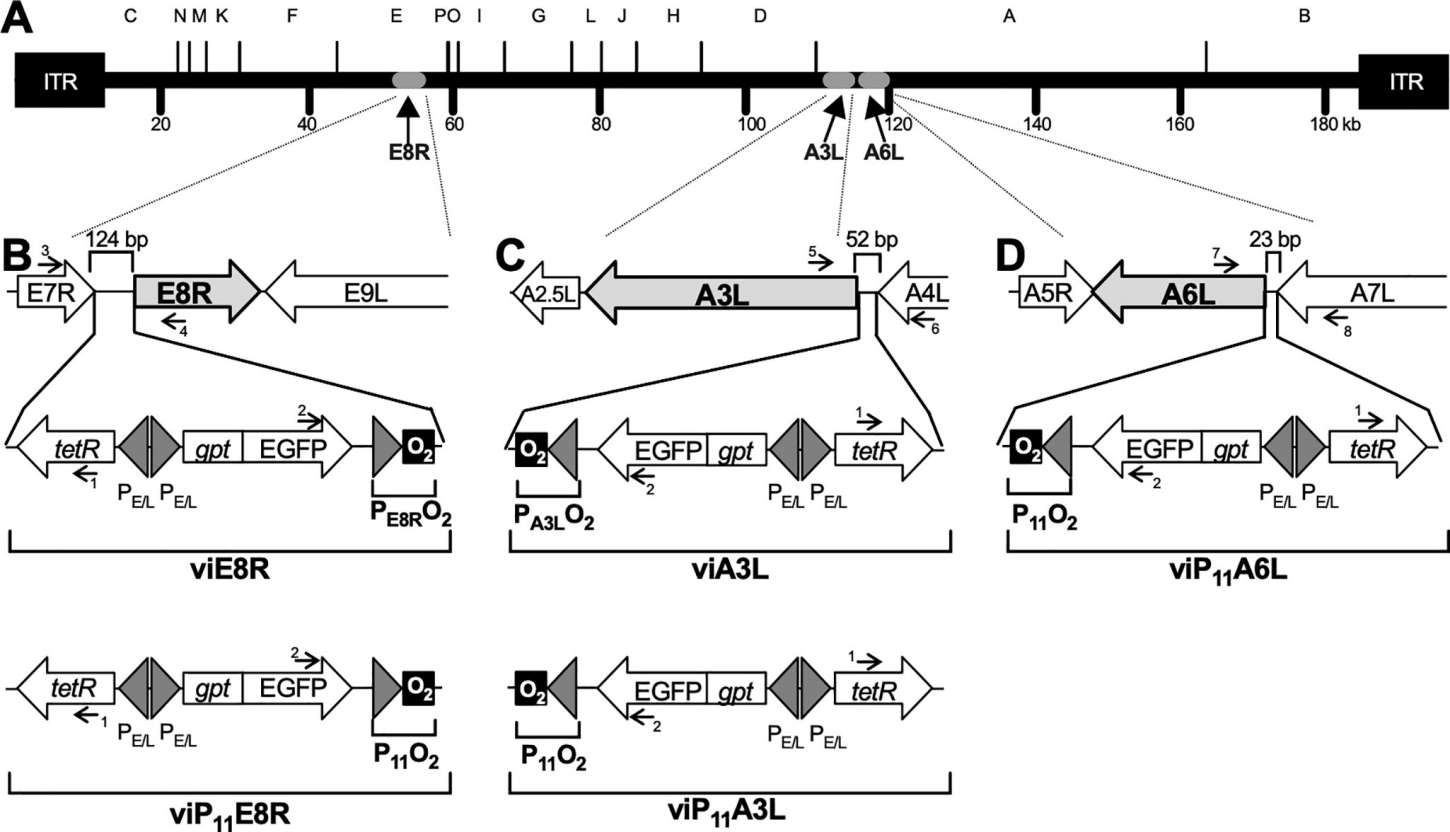
Genomic organization of the VACVs inducibly expressing the E8R, A3L, or A6L genes. (**A**) Genome of the WR strain of VACV showing *Hin*dIII restriction fragments A through P and the location of the E8R, A3L, and A6L genes. Cassettes containing the putative E8R (P_E8R_) or A3L (P_A3L_) promoters, or the P_11_ promoter, followed by the *tet* operator (O_2_) were inserted upstream of the E8R, A3L, or A6L genes to generate the recombinant VACVs viE8R (**B**), viA3L (**C**), and viP_11_A6L (**D**), respectively. Replacement of P_E8R_ and P_A3L_ promoters with P_11_ resulted in viP_11_E8R (**B**, lower panel) and viP_11_A3L (**C**, lower panel). The cassettes also contain the *tetR* gene and the *gpt*-EGFP fusion gene under back-to-back synthetic early/late VACV promoters (P_E/L_). Arrows with numbers indicate primers (Table 2) used to amplify specific genomic regions for characterization of the viruses. ITR, inverted terminal repeat. Panels B-D are not drawn to scale.

Replication-inducible recombinant VACVs were designed by expressing the *tetR* gene under a constitutive VACV promoter and incorporating *tet*O_2_ immediately downstream from the promoters directing the expression of the E8R, A3L, or A6L genes (Fig 1). VACV late promoter sequences contain an A/T-rich stretch of approximately 20 bp, a 6 bp spacer region, and a highly conserved TAAAT(A/G) transcriptional initiator element [52]. The intergenic sequences upstream from the E8R and A3L genes are 124 and 52 bp, respectively. Since they are expected to contain only the promoters for the E8R and A3L genes, the O_2_ operator was inserted immediately after the putative late transcriptional initiator sequence (TAAATA) of these genes as shown in Fig 1B and 1C, and in Table 1. The intergenic region upstream of the A6L gene is only 23 bp, and since it is shorter than the typical late poxvirus promoter, it is likely that a segment of the A6L promoter is located within the upstream A7L gene. The intergenic region contains more than one putative late transcriptional initiator sequence, so the promoter boundaries could not be identified with confidence. Therefore, the well-characterized F17R (P_11_) late promoter, which has been used successfully as a *lac*-responsive promoter [53], was used to control the transcription of the A6L gene (Fig 1D and Table 1). The resulting recombinant VACVs (viE8R for VACV inducible E8R, viA3L for VACV inducible A3L, and viP_11_A6L for VACV inducible A6L using the P_11_ promoter) were expected to replicate only in the presence of tetracyclines such as DOX.

**Table 1:** Sequence of the putative natural promoters and the *tet* operator-controlled promoters used to generate the replication-inducible VACVs.

Promoter^a^	Sequence^b^
F17R (P_11_) natural	ATTTAGAATATATGTATGTAAAAATATAGTAGAATTTCATTTTGTTTTTTTCTATGCTATAA**ATG**
P_11_O_2_^c^	ATATAGTAGAATTTCATTTTGTTTTTTTCTATGCTATAAATATCCCTATCAGTGATAGAGACGGCCG**ATG**
E8R natural	GTATAATCCCATTCTAATACTTTAACCTGATGTATTAGCATCTTATTAGAATATTAACCTAACTAAAAGACATAACATAAAAACTCATTACATAGTTGATAAAAAGCGGTAGGATATAAATATT**ATG**
P_E8R_O_2_	GTATAATCCCATTCTAATACTTTAACCTGATGTATTAGCATCTTATTAGAATATTAACCTAACTAAAAGACATAACATAAAAACTCATTACATAGTTGATAAAAAGCGGTAGGATATAAATATCCCTATCAGTGATAGAGACGGCCGATG
A3L natural	TAAGATTGGATATTAAAATCACGCTTTCGAGTAAAAACTACGAATATAAATA**ATG**
P_A3L_O_2_	TAAGATTGGATATTAAAATCACGCTTTCGAGTAAAAACTACGAATATAAATATCCCTATCAGTGATAGAGACGGCCGATG
A6L natural	ACAACTAAATCTGTAAATAAATA**ATG**

^a^ The putative natural promoters were defined as the intergenic regions upstream from the genes. The O_2_-controlled promoters are based on the putative natural promoters or the F17R (P_11_) promoter.

^b^ The promoter sequences are shown with the putative late transcriptional initiator element sequences boxed, the start codons bolded, and the O_2_ operator sequences underlined.

^c^ The P_11_O_2_ promoter was used to control expression of E8R in viP_11_E8R, A3L in viP_11_A3L, and A6L in viP_11_A6L.

A series of cloning steps were used to build the transfer vectors based on existing plasmids, designed synthetic DNA sequences, and PCR cloning. The final transfer vectors contained the selectable *Escherichia coli* xanthine-guanine phosphoribosyl transferase (*gpt*) gene and the screening marker enhanced green fluorescent protein (EGFP) gene as a fusion gene (*gpt*-EGFP) under the control of the synthetic strong early/late VACV promoter P_E/L_ [54] (thus allowing both selection and screening of recombinants using a single VACV promoter), the repressor gene *tetR* under another (back-to-back) P_E/L_ promoter, a *tet*-responsive promoter (P_E8R_O_2_, P_A3L_O_2_, or P_11_O_2_) to control the expression of the target genes, and left and right recombination sequences (the first 600 bp to the left and to the right of the intergenic regions shown in Fig 1B–1D) to direct the precise insertion of the genetic elements contained in each cassette by homologous recombination. The recombinant viruses were successfully constructed and plaque purified in the presence of mycophenolic acid selection medium and DOX. High-titer stocks lacked EGFP^-^ plaques that would represent unstable recombinant VACVs or residual parental (wild-type) virus. In addition, PCR analysis of viral DNA purified from high-titer stocks with multiple primers spanning the regions of interest (Fig 1B–1D and Table 2) confirmed the overall genetic organization of these regions in each recombinant VACV.

**Table 2:** Primers used to amplify wild-type and recombinant VACV genomic regions.

Primer	Gene	Sequence (5’ to 3’)
1	*tetR*	GACGCCTTAGCCATTGAGAT
2	EGFP	ACAACCACTACCTGAGCACC
3	E7R	TCTCCGCACATGGAACTCAT
4	E8R	CAGAGAACGATCCATTAGCA
5	A3L	GATGCTACTTCGTCGATGGA
6	A4L	CAAATCCAGGAGCAGCATCT
7	A6L	TATCAACATCTGATGCGCT
8	A7L	ATGTTATTGCGTCTGATGCC
9	I8R (forward)	ATTTTCCAATTCCGTAGGTAAACGA
10	I8R (reverse)	TGATCATGCTCATGAACTTCGTCTA
11	A6L (forward)	TAAATACGGCCGATGGACAAACTTAG
12	A6L (reverse)	ACGATAGCTAGCTTAGAATTTATACG
13	A3L (forward)	TAAATACGGCCGATGGAAGCCGTG
14	A3L (reverse)	CATAATGCTAGCCTAAAATAGTTC

### viP_11_A6L forms plaques in the presence of DOX and produces abortive infections in the absence of DOX

**Fig 2:**
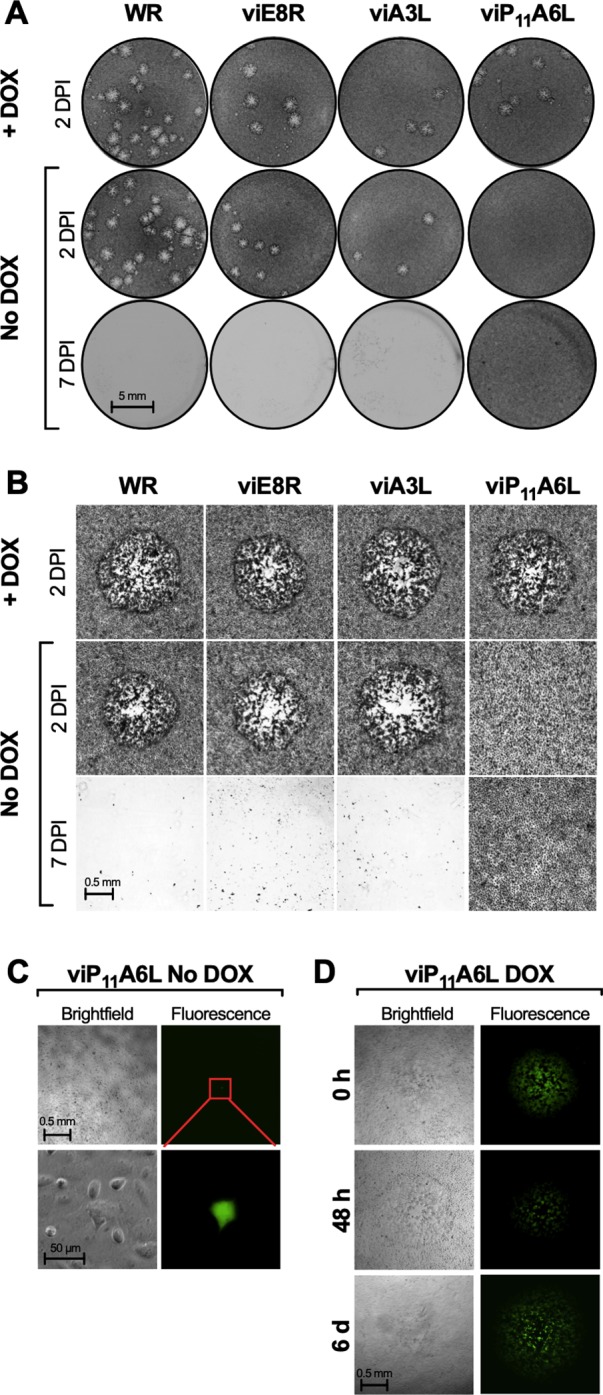
viP_11_A6L forms plaques only in the presence of DOX. BS-C-1 cell monolayers were infected with the indicated VACVs at approximately 5–20 PFU/well in the absence or presence of 1 μg/ml DOX and cells were stained with crystal violet 2 or 7 DPI (**A** and **B**) or imaged by brightfield (phase) and fluorescence microscopy (**C** and **D**). (**A**) Image of representative wells showing the plaque phenotypes. (**B**) Representative brightfield microscopic images of stained cells showing plaques, when present. WR refers to VACV WR (parental strain). In the absence of DOX only single EGFP^+^ cells were observed 2 DPI for viP_11_A6L (**C**), and under higher magnification, EGFP expression was contained to single cells and was the only indication of infection (red inset), suggesting abortive infections. When DOX was added at the time of infection (0 h), 48 h, or 6 days after infection (**D**), plaques were visible 2 days later (2, 4, or 8 DPI, respectively). Data is representative of two separate experiments.

The ability of the putative inducible viruses to replicate in the absence or presence of inducer was first investigated by performing standard plaque assays in BS-C-1 cells, either in the absence or presence of DOX (1 μg/ml), followed by crystal violet staining 2 or 7 days post-infection (DPI). Isolated plaques that formed 2 DPI in the presence of DOX by viE8R, viA3L, and viP_11_A6L were typical (Fig 2A and 2B) and identical to wild-type WR plaques in size. In the absence of DOX, viE8R and viA3L formed distinct plaques 2 DPI (Fig 2A and 2B), suggesting that control of E8R and A3L gene expression was unsuccessful. However, no plaques could be detected 2 DPI for viP_11_A6L in the absence of DOX (Fig 2A and 2B). Moreover, plaques were not observed in cells infected with viP_11_A6L in the absence of DOX even 7 DPI (Fig 2A), when the entire cell monolayer infected with WR, viE8R, or viA3L displayed extensive cytopathic effects (CPE) and therefore destruction of the monolayer, resulting in a lack of crystal violet staining.

Plaque formation in BS-C-1 cells was also investigated in unfixed cells by brightfield and fluorescence microscopy. Under fluorescence microscopy, viE8R and viA3L formed typical plaques both in the absence and presence of DOX. However, with viP_11_A6L, only single EGFP^+^ cells could be detected in the absence of DOX (Fig 2C). The frequency of these cells corresponded roughly to the number of plaques obtained in the presence of DOX, where EGFP^+^ plaques were observed (Fig 2D). Under high magnification, the single EGFP^+^ cells appeared normal and there was no evidence of EGFP expression in the neighboring cells (Fig 2C). Taken together, these observations are indicative of abortive infections. In addition, detection of high levels of EGFP expression in these abortively-infected cells suggests that late gene expression from the P_E/L_ promoter was not compromised in the absence of DOX. When DOX was added to these abortively-infected cells 2, 4, or 6 DPI, replication resumed and plaques were visible 2 days later (Fig 2D), indicating that transcription of the A6L gene was sufficient to allow the resumption and completion of the replication cycle. However, plaques were not detected when DOX was added 8 or 10 DPI, demonstrating that resumption and completion of the replication cycle was only allowed up to 6 DPI.

### viP_11_A6L plaque size and replication are indistinguishable from WR in the presence of DOX

**Fig 3:**
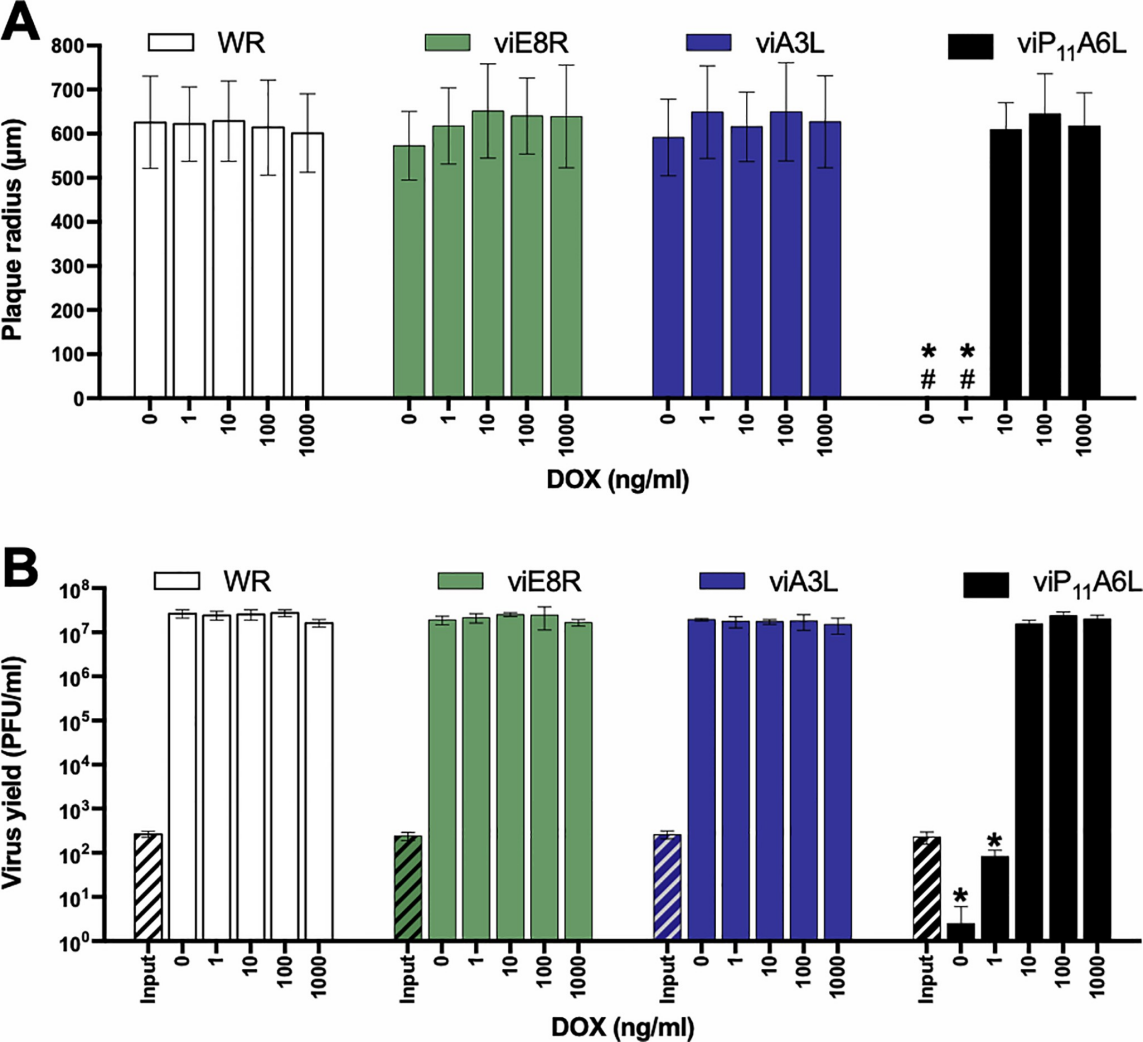
viP_11_A6L replicates indistinguishably from WR in the presence of DOX. (**A**) The effect of DOX on plaque size was examined by infecting BS-C-1 cell monolayers with the VACVs in the absence or presence of multiple concentrations of DOX. At 36 hpi, cells were stained with crystal violet and the size (radius) of approximately 20 representative isolated plaques was measured (# indicates absence of plaques). (**B**) The effect of DOX on virus replication was examined by infecting BS-C-1 cell monolayers with the indicated VACVs at an MOI of 0.01. Cells were collected immediately to determine input titer (hatched bars) or after 48 h in the absence or presence of multiple concentrations of DOX to determine virus yield (solid bars). Titers were determined on BS-C-1 cells in the presence of 1 μg/ml DOX. The data shown represent the mean viral yields from triplicate samples assayed in duplicate. Error bars indicate standard deviation. An asterisk indicates statistically significant differences (*p* < 0.05 by two-way ANOVA followed by Tukey’s multiple comparisons test) between WR and the inducible viruses at a given DOX concentration. Data is representative of two separate experiments.

The size of the plaques formed by WR and the inducible viruses in the absence or presence of multiple concentrations of DOX was analyzed by plaque assay in BS-C-1 cells. The radius of plaques formed by WR was not affected by the presence of DOX, and viE8R and viA3L formed plaques comparable in size to WR, even in the absence of DOX (Fig 3A). In the absence or presence of 1 ng/ml DOX, viP_11_A6L did not form plaques. In the presence of 10, 100, and 1000 ng/ml DOX, viP_11_A6L formed typical plaques that were indistinguishable from plaques formed by WR.

The replication of each inducible virus was assessed in BS-C-1 cells infected at a multiplicity of infection (MOI) of 0.01 in the absence or presence of multiple concentrations of DOX by determining virus yield 48 hpi. As expected, the wild-type WR strain replicated equally in the absence or presence of DOX (Fig 3B). Additionally, viE8R and viA3L replicated indistinguishably from WR both in the absence and presence of DOX. viP_11_A6L yield was below the input level at 0 and 1 ng/ml of DOX. More importantly, viP_11_A6L yield at ≥10 ng/ml of DOX was indistinguishable from WR, demonstrating that viP_11_A6L replicates to wild-type levels under these conditions.

### VACV expressing A3L under the P_11_ promoter is replication-inducible and replicates indistinguishably from wild-type VACV in the presence of DOX

The putative natural promoter sequences present in the upstream intergenic regions were used to control the expression of the E8R and A3L genes in viE8R and viA3L, respectively (Fig 1 and Table 1). Since viE8R and viA3L replicated even in the absence of tetracyclines, indicating a lack of control of E8R or A3L gene expression, respectively, the natural promoters of the A3L and E8R genes were replaced with the inducible late P_11_O_2_ promoter used to develop viP_11_A6L (Table 1). This was accomplished by replacing the P_E8R_O_2_ and P_A3L_O_2_ promoters in the transfer vectors (Fig 1B and 1C) with the P_11_O_2_ promoter sequence (Table 1). New recombinant VACVs were generated expressing E8R (viP_11_E8R) or A3L (viP_11_A3L) under P_11_O_2_.

**Fig 4:**
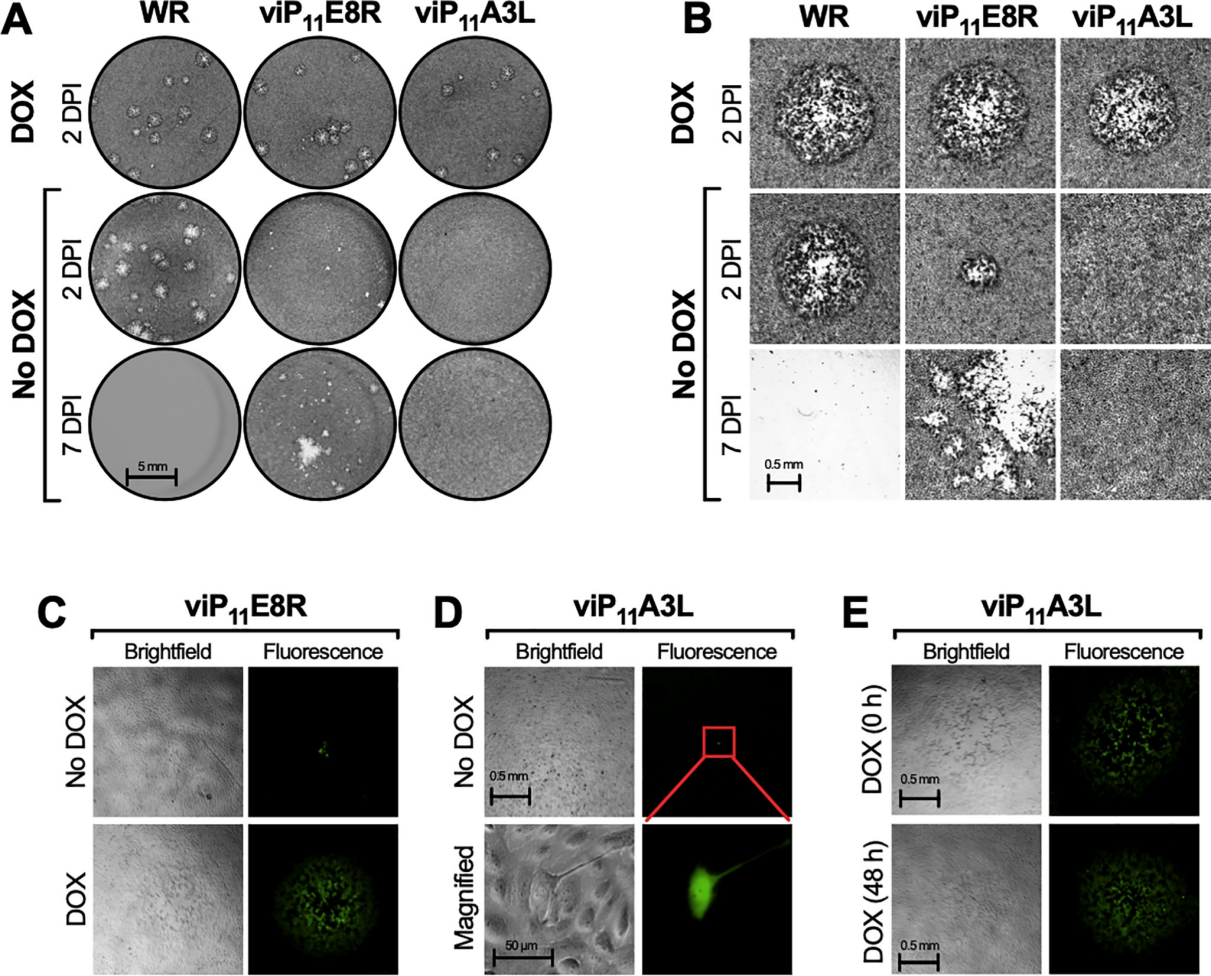
viP_11_A3L does not form plaques and causes abortive infections in the absence of DOX. BS-C-1 cell monolayers were infected with the indicated VACVs at approximately 5–20 PFU/well in the absence or presence of 1 μg/ml DOX and cells were stained with crystal violet 2 or 7 DPI (**A** and **B**) or imaged by brightfield (phase) and fluorescence microscopy (**C**, **D**, and **E**). **(A)** Image of representative wells showing the plaque phenotypes. (**B**) Representative brightfield microscopic images of stained cells showing plaques, when present. (**C**) In the absence of DOX smaller plaques formed 2 DPI with viP_11_E8R. (**D**) In the absence of DOX, EGFP expression was contained to single viP_11_A3L-infected cells and was the only indication of infection. (**E**) When DOX was added at the time of infection or 48 h after infection, plaques were visible 2 and 4 days later, respectively. Data is representative of two separate experiments.

Both viP_11_E8R and viP_11_A3L produced plaques similar to WR in the presence of DOX (Fig 4A and 4B). In the absence of DOX, no plaques were detected with viP_11_A3L at 2 or 7 DPI, while viP_11_E8R formed noticeably smaller plaques 2 DPI and caused additional CPE by 7 DPI (Fig 4A and 4B). Under fluorescence microscopy, viP_11_E8R formed small plaques in the absence of tetracyclines and typical plaques in the presence of DOX (Fig 4C). However, only single EGFP^+^ cells could be detected in the absence of DOX with viP_11_A3L (Fig 4D). Under high magnification, the single EGFP^+^ cells appeared normal and there was no evidence of EGFP expression in the neighboring cells (Fig 4D), indicating abortive infections. When DOX was added to these abortively-infected cells 2, 4, or 6 DPI, replication was allowed to resume and plaques were visible 2 days later (Fig 4E shows results for addition of DOX 48 h post-infection). However, as seen with viP_11_A6L, viP_11_A3L plaques were not detected when DOX was added 8 or 10 DPI.

**Fig 5:**
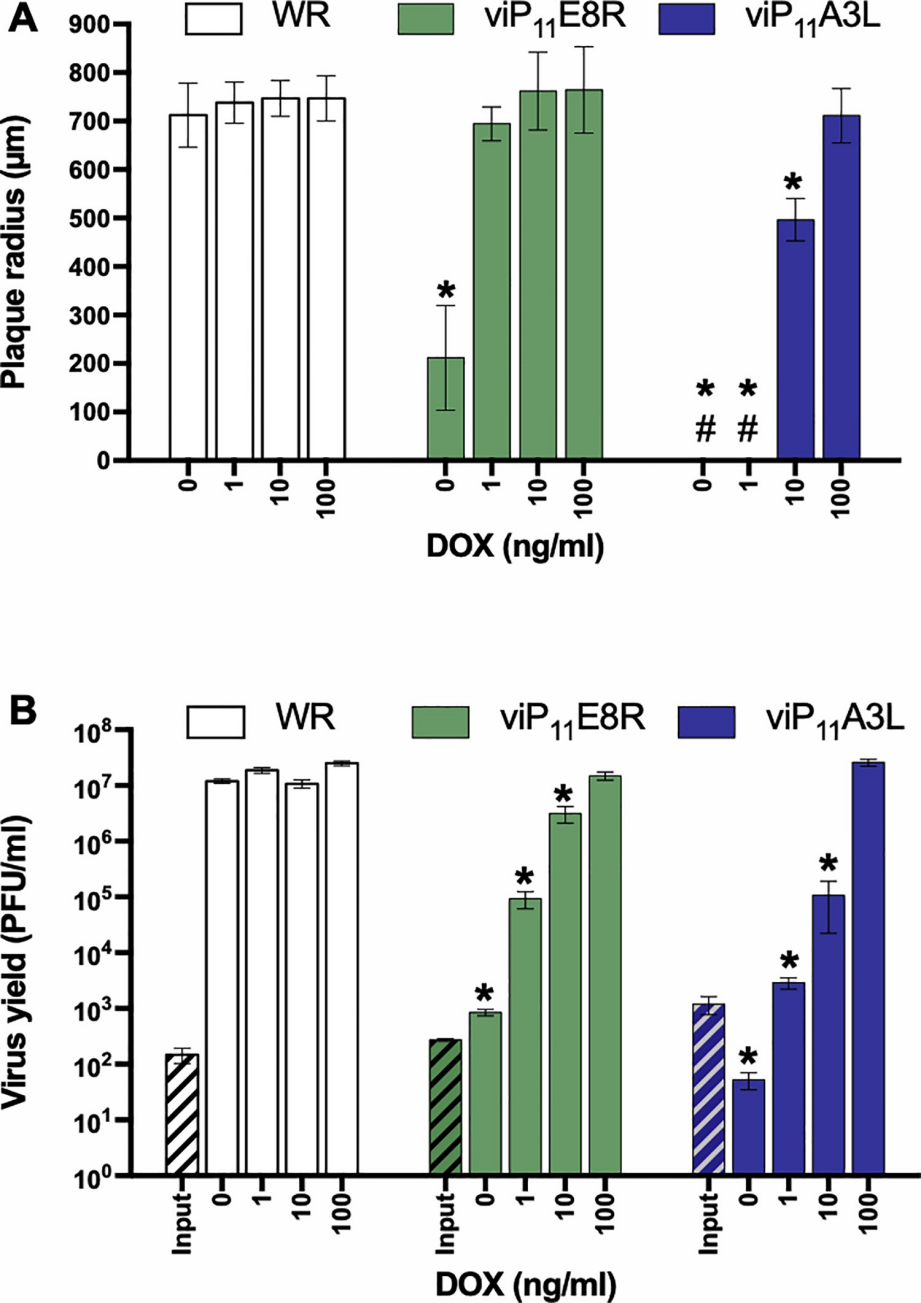
viP_11_A3L replicates indistinguishably from wild-type VACV in the presence of DOX. (**A**) The effect of DOX on plaque size was examined by infecting BS-C-1 cell monolayers with the VACVs in the absence or presence of multiple concentrations of DOX. At 36 hpi, cells were stained with crystal violet and the size (radius) of approximately 20 representative isolated plaques was measured (# indicates absence of plaques). (**B**) The effect of DOX on virus replication was examined by infecting BS-C-1 cell monolayers with the indicated VACVs at an MOI of 0.01. Cells were collected immediately to determine input titer (hatched bars) or after 48 h in the absence or presence of multiple concentrations of DOX to determine virus yield (solid bars). Titers were determined on BS-C-1 cells in the presence of 1 μg/ml DOX. The data shown represent the mean viral yields from triplicate samples assayed in duplicate. Error bars indicate standard deviation. An asterisk indicates statistically significant differences (*p* < 0.05 by two-way ANOVA followed by Tukey’s multiple comparisons test) between WR and the inducible viruses at a given DOX concentration.

The size of plaques formed by viP_11_E8R and viP_11_A3L was assessed by standard plaque assay in BS-C-1 cells in the absence or presence of multiple concentrations of DOX. In the presence of ≥1 ng/ml DOX, viP_11_E8R formed typical plaques that were comparable to WR (Fig 5A). However, viP_11_E8R formed plaques even in the absence of DOX, although they were significantly smaller than WR plaques. No plaques were detected in viP_11_A3L-infected cell monolayers in the absence or presence of 1 ng/ml DOX. In the presence of 10 ng/ml DOX, viP_11_A3L formed plaques that were smaller when compared to WR. Notably, viP_11_A3L produced plaques comparable in size to WR in the presence of 100 ng/ml DOX.

The replication of viP_11_E8R and viP_11_A3L was assessed in BS-C-1 cells infected with the VACVs at an MOI of 0.01 in the absence or presence of multiple concentrations of DOX (Fig 5B). As expected, viP_11_E8R replicated only minimally in the absence of tetracyclines, and in a dose-dependent manner when DOX was added, reaching wild-type levels at 100 ng/ml DOX. There was no evidence of viP_11_A3L replication in the absence of DOX, although some level of replication was detected at 1 ng/ml, despite the fact that no plaques were observed at this concentration (Fig 5A and 5B). At 10 ng/ml DOX, viP_11_A3L replicated to intermediate levels, and more importantly, the yield at 100 ng/ml DOX was indistinguishable from WR, demonstrating that viP_11_A3L replicates to wild-type levels under these conditions. Interestingly, viP_11_A6L required only 10 ng/ml DOX to replicate to wild-type levels (Fig 3), perhaps because it is needed in lower amounts to allow full replication.

### Transient complementation allows replication of viP_11_A6L and viP_11_A3L in the absence of DOX

**Fig 6:**
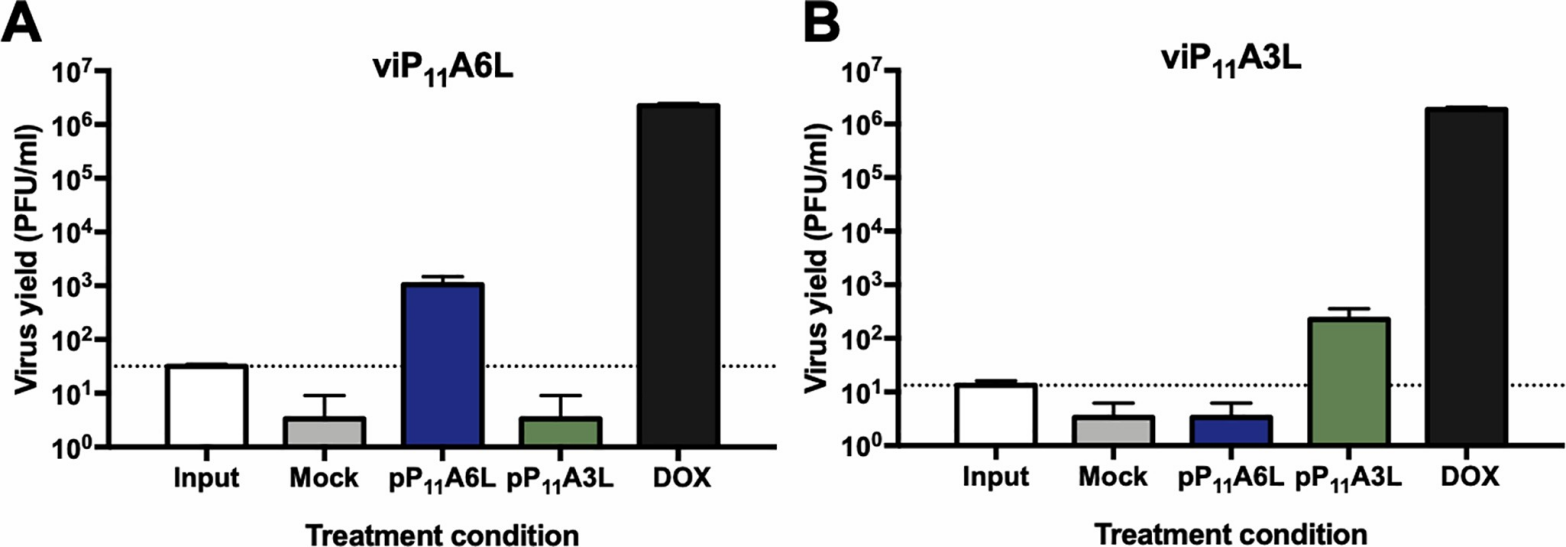
Transient complementation allows viP_11_A6L and viP_11_A3L replication in the absence of DOX. BS-C-1 cell monolayers were infected with viP_11_A6L (**A**) or viP_11_A3L (**B**) at an MOI of 0.01 in the absence of DOX and transfected with plasmids expressing the A6L (pP_11_A6L) or A3L (pP_11_A3L) genes under the constitutive VACV P_11_ promoter, or no plasmid (mock). Infections were also performed in the presence of 1 μg/ml DOX (DOX). Cells were collected immediately after infection (input, dotted line) or 2 DPI. Virus yield was determined by plaque assay on BS-C-1 cells in the presence of 1 μg/ml DOX. The data shown represent the mean viral yields from triplicate samples assayed in duplicate. Error bars indicate standard deviation. Data are representative of two separate experiments.

To confirm that replication of viP_11_A6L and viP_11_A3L is dependent on the expression of the A6L or A3L genes, respectively, transient complementation assays were performed in the absence of DOX. BS-C-1 cells were infected with viP_11_A6L or viP_11_A3L and transfected with plasmids expressing the A6L or A3L genes constitutively under the VACV P_11_ promoter (pP_11_A6L or pP_11_A3L), or no plasmid (mock). Complementation was assessed by measuring virus yield in the absence or presence of the complementing plasmids. Cells infected with viP_11_A6L and transfected with pP_11_A6L yielded approximately a two-log increase in virus titers when compared to cells transfected with pP_11_A3L or no plasmid, although yield was not as high as virus grown in the presence of DOX expressing the A6L gene directly from the genome (Fig 6A). Similar results were seen in cells infected with viP_11_A3L in the presence of the complementing plasmid pP_11_A3L (Fig 6B).

### viP_11_A6L replicates *in vivo* in the presence of DOX

**Fig 7:**
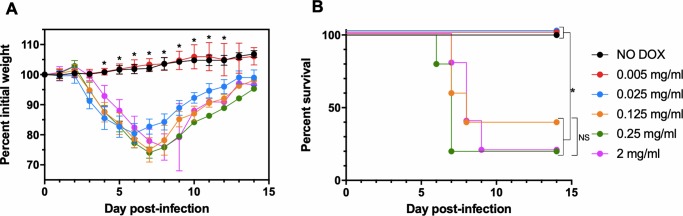
viP_11_A6L causes weight loss in mice in the presence of DOX. Groups of female CB6F_1_/J mice (n = 5) were inoculated intranasally with ~5 × 10^4^ PFU viP_11_A6L in the absence or presence of different concentrations of DOX in drinking water. Weight and mortality were assessed daily. Animals were euthanized if weight loss was ≥ 25%. (**A**) Mean group weights are displayed as a percentage of group weight on day 0. An asterisk represents statistically significant differences (*p* < 0.01) determined using one-way ANOVA followed by Dunnett’s multiple comparisons test comparing NO DOX to all other groups at each day post-infection. Error bars indicate standard deviation. (**B**) Percent survival is shown. An asterisk represents statistically significant differences (*p* < 0.05) by log-rank (Mantel-Cox) test for differences in survival adjusted for multiple comparisons using the Bonferroni *post-hoc* test. NS = not significant.

We next assessed replication of viP_11_A6L *in vivo*, since it replicated *in vitro* to wild-type levels at lower concentrations of DOX (Fig 3) when compared to viP_11_A3L (Fig 5). Groups of five female CB6F_1_/J mice were inoculated intranasally with ~5 × 10^4^ PFU viP_11_A6L, a dose expected to cause approximately 50% mortality [55]. Mice were given either normal drinking water (NO DOX), or DOX dissolved in drinking water at either 0.005, 0.025, 0.125, 0.25, or 2 mg/ml, offered *ad libitum* beginning 1 day before inoculation and continued through the end of the study. We have previously administered 2 mg/ml DOX in drinking water to successfully induce expression from the P_11_O_2_ promoter *in vivo* [56] and wanted to determine if lower concentrations of DOX would also result in viral replication sufficient to cause weight loss and mortality similar to 2 mg/ml. Infected animals were weighed daily and euthanized if weight loss was ≥ 25%. All animals given normal drinking water (NO DOX) or 0.005 mg/ml DOX did not lose weight or display any other clinical signs, and survived with significantly higher mean group weights compared to all other groups 4–12 DPI (Fig 7). Infected animals given 0.025 mg/ml DOX lost weight but survived, with mild clinical signs such as ruffled fur. In contrast, all infected animals given ≥ 0.125 mg/ml DOX lost weight and displayed clinical signs including ruffled fur, hunched posture, and decreased activity. Some (12/25) succumbed to infection or were euthanized (weight loss ≥ 25%). Survival was significantly different when comparing NO DOX, 0.005, and 0.025 mg/ml DOX with all other groups, whereas it was not significantly different between 0.125, 0.25, and 2 mg/ml. A concentration of 0.125 mg/ml DOX was used in subsequent mouse studies because it was the lowest concentration tested that resulted in weight loss and mortality similar to 2 mg/ml DOX.

### viP_11_A6L causes weight loss and mortality similar to wild-type VACV in mice treated with DOX

**Fig 8:**
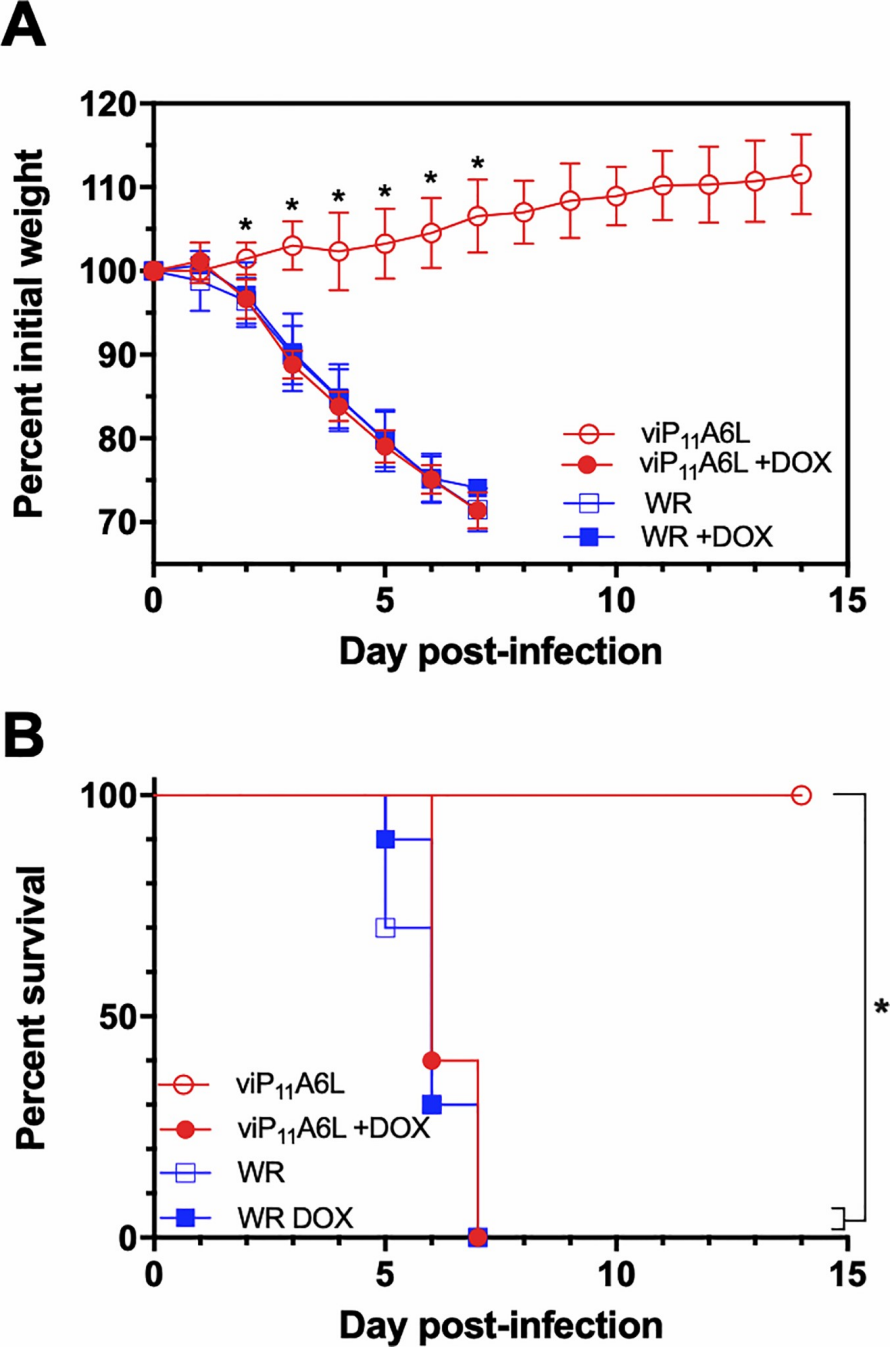
viP_11_A6L causes weight loss and mortality similar to WR in the presence of DOX. Groups of female CB6F_1_/J mice (n = 10) were inoculated intranasally with ~2 × 10^6^ PFU viP_11_A6L or WR in the absence or presence of DOX. Weight and mortality were assessed daily. Animals were euthanized if weight loss was ≥ 25%. Mean group weights as a percentage of group weight on Day 0 (**A**), or percent survival (**B**) are shown. Asterisks indicate statistical significance (*p* < 0.01) by one-way ANOVA followed by Sidak’s multiple comparisons test (**A**), or by log-rank (Mantel-Cox) test between indicated groups adjusted for multiple comparisons using the Bonferroni *post-hoc* test (**B**). Error bars indicate standard deviation.

To compare replication of the inducible viP_11_A6L to the wild-type parental WR, groups of 10 CB6F_1_/J mice were infected intranasally with a lethal dose of ~2 × 10^6^ PFU virus (approximately 40 times the 50% lethal dose) [55] in the absence or presence of 0.125 mg/ml DOX in drinking water. In the absence of DOX, all mice inoculated with viP_11_A6L survived infection without weight loss or other clinical signs, with significantly higher mean weights than all other groups from 2–8 DPI. In the presence of DOX, viP_11_A6L-infected mice exhibited weight loss, clinical symptoms (including ruffled fur, hunched posture, and decreased activity), and mortality indistinguishable from WR (Fig 8).

### viP_11_A6L viral loads in ovaries are equivalent to WR levels in the presence of DOX and undetectable in the absence of DOX

**Fig 9:**
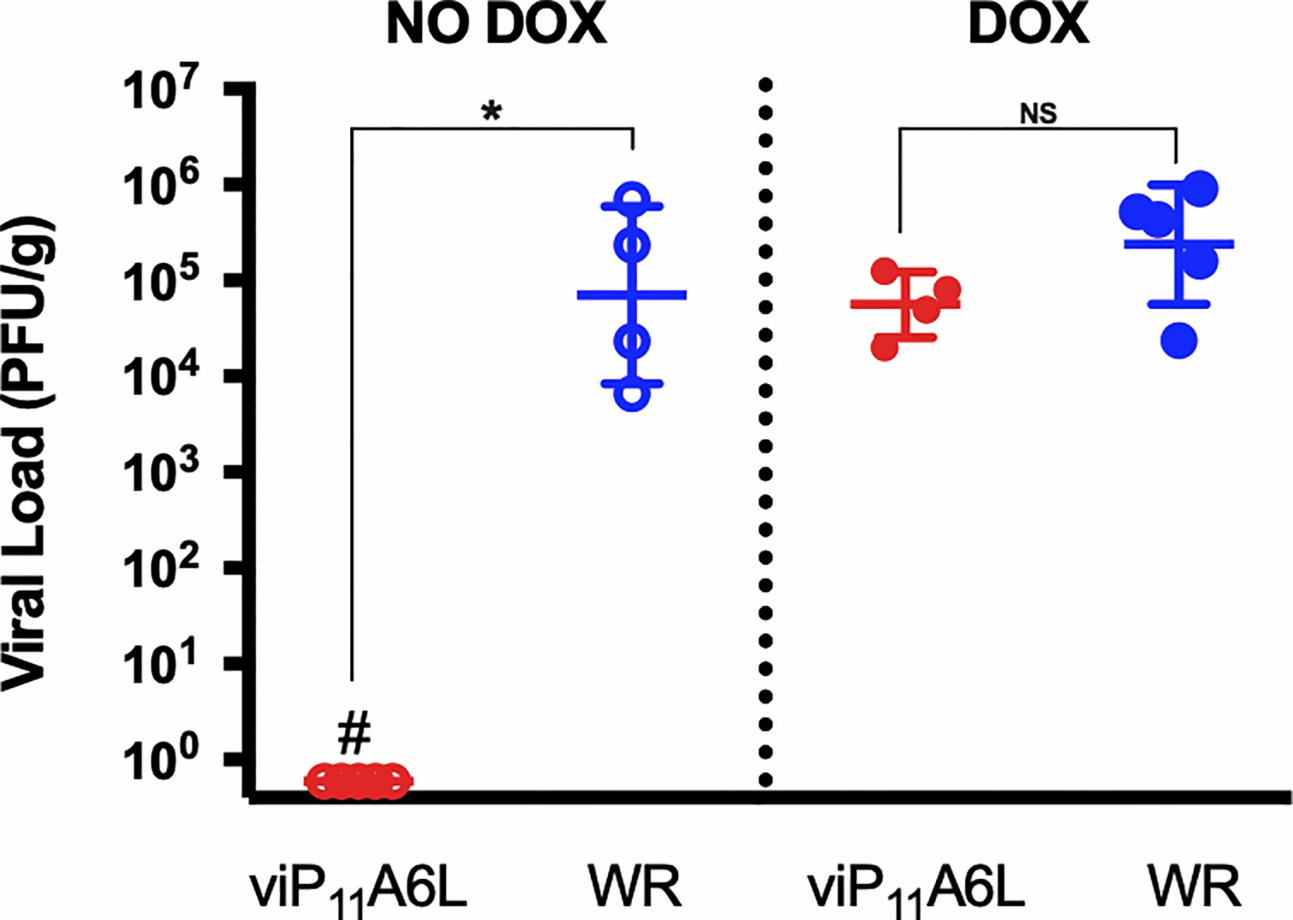
viP_11_A6L replicates indistinguishably from wild-type VACV in mice treated with DOX. Groups of five female CB6F_1_/J were inoculated intraperitoneally with ~2 × 10^6^ PFU viP_11_A6L or WR in the presence or absence of DOX in drinking water. Mice were euthanized 6 DPI, and ovaries collected and processed. Ovarian homogenates were added to BS-C-1 cells in the presence of 1 μg/ml DOX to determine viral loads (# indicates absence of plaques via plaque assay). Asterisk indicates statistically significant differences (*p* < 0.01) by Mann-Whitney test between groups in each DOX treatment. NS = not significant.

To compare disseminated replication of the inducible viP_11_A6L to the parental WR in tissue, groups of five CB6F_1_/J female mice were infected intraperitoneally with ~2 × 10^6^ PFU viP_11_A6L or WR, in the absence or presence of 0.125 mg/ml DOX in drinking water. Viral loads in ovaries were determined by plaque assay 6 DPI (Fig 9), since VACV replicates to high titers in ovaries after intraperitoneal inoculation [15]. Virus was not detected in ovaries from mice infected with viP_11_A6L in the absence of DOX. Conversely, viral loads in ovaries of mice infected with viP_11_A6L in the presence of DOX were indistinguishable from those of mice infected with WR.

## Discussion

VACV was first used as a live vaccine to successfully eradicate smallpox worldwide [1]. Since then, VACV has been used for the development of recombinant live vaccines due to the ease of genetically manipulating its large dsDNA genome while retaining infectivity, its heat stability, and relatively low cost of production [57, 58]. Successful examples include a recombinant VACV expressing the rabies virus glycoprotein (V-RG) that has been used in Europe and North America against sylvatic rabies [59], and a VACV expressing rinderpest virus glycoproteins provided long-term sterilizing immunity in cattle [60]. Currently, a number of clinical trials are underway for animal and human vaccines, immunotherapies, and oncolytic therapies based on replication-competent VACV vectors [61, 62]. However, the potential for severe adverse reactions is a concern, especially in individuals with predisposing conditions. Replication-deficient VACVs such as MVA address these safety issues, although they are not as immunogenic as replication-competent VACVs [63], grow to lower titers in cell culture [12], and cannot be used for oncolytic therapies. Here, we generated replication-inducible VACVs using elements of the *tet* operon to allow tight control of viral replication with tetracycline antibiotics. Importantly, these VACV vectors replicate at wild-type levels in the presence of inducer, and thus are expected to maintain their full immunogenic and oncolytic potential.

The replication-inducible VACVs were designed to control the expression of genes encoding core or virion morphogenesis proteins. The O_2_ operator sequence was placed immediately downstream from the late transcriptional initiator elements of the E8R and A3L promoters in viE8R and viA3L, respectively. In viP_11_A6L, a late P_11_ promoter and O_2_ operator sequence were used to control A6L gene expression since the natural promoter could not be identified with confidence. In addition, the *tetR* gene was constitutively expressed at high levels in all viruses using a synthetic early/late VACV promoter (P_E/L_).

We showed that viP_11_A6L does not form plaques in the absence of DOX, even when cells were incubated for 7 days. Singly-infected cells could be detected by expression of the EGFP reporter protein 2 DPI, and the neighboring cells did not show any evidence of infection such as CPE or EGFP expression. High levels of EGFP expression in these abortively-infected cells suggest that gene expression from the P_E/L_ promoter was not compromised in the absence of DOX. In the presence of DOX, viP_11_A6L formed typical plaques, even when DOX was added 2, 4, or 6 DPI, indicating that the virus growth cycle resumed once A6L gene expression was allowed. Replication did not resume when DOX was added 8 or 10 DPI, possibly because the abortively-infected cells or the virus life cycle were compromised this late after infection. Additionally, viP_11_A6L did not form plaques in the absence or presence of 1 ng/ml DOX, and plaque sizes were indistinguishable from wild-type VACV at ≥10 ng/ml DOX. viP_11_A6L replicated minimally at 1 ng/ml and virus yield at ≥10 ng/ml DOX was indistinguishable from WR, demonstrating that viP_11_A6L replicates to wild-type levels under these conditions.

Since viE8R and viA3L replicated even in the absence of tetracyclines, it is possible that control of the native E8R and A3L promoters was unsuccessful because their sequences were not identified correctly. Thus, the putative natural E8R and A3L promoter sequences were replaced with the P_11_ (F17R) promoter sequence, used successfully in viP_11_A6L, to generate viP_11_E8R and viP_11_A3L. However, in the absence of tetracyclines viP_11_E8R still formed plaques (although noticeably smaller) and showed evidence of replication. Conversely, no plaques were detected in viP_11_A3L-infected cell monolayers in the absence or presence of 1 ng/ml DOX, and plaques comparable in size to WR were formed in the presence of 100 ng/ml DOX. Notably, there was no evidence of viP_11_A3L replication in the absence of DOX, and virus yield at 100 ng/ml DOX was indistinguishable from WR. Additionally, transient complementation confirmed that inducible replication of viP_11_A3L and viP_11_A6L was achieved through control of A3 and A6 expression. viP_11_A6L was further tested *in vivo*, where weight loss and mortality were observed after intranasal infection in the presence of only 0.125 mg/ml DOX in drinking water. Importantly, mice inoculated with viP_11_A6L in the presence of DOX exhibited similar levels of weight loss, mortality, and viral replication as mice inoculated with wild-type (WR) VACV, but did not result in any clinical signs in the absence of DOX.

The E8R gene has been studied extensively, although its specific function remains unclear [37–39]. In our study, when the E8R gene was placed under the control of *tet* operon elements, the virus replicated both in the absence and presence of tetracyclines. Interestingly, viP_11_E8R plaques formed in the absence of inducer were significantly smaller than wild-type VACV plaques. This suggests leaky expression of E8R in the absence of tetracyclines, or that the E8R gene is not essential for viral replication *in vitro*.

The function of A3 has been studied previously using temperature-sensitive mutants where it was found that virions formed at non-permissive temperatures were abnormal in shape, had substantially reduced infectivity, and reached transcription levels less than 2% of the wild-type virus, thus indicating A3 plays a critical role in virion morphogenesis [44]. Additionally, an inducible A3L virus has been generated using *lac* operon elements in a system where the bacteriophage T7 RNA polymerase is expressed under a late promoter controlled by a *lac* operator, and the A3L gene is placed under a T7 promoter also controlled by a *lac* operator [64]. In the presence of inducer (IPTG) the virus formed significantly smaller plaques, and in the absence of inducer, abnormal immature virions accumulated within the cytoplasm and no plaques were observed, demonstrating that A3 is required for normal virion formation. In our study, we also successfully demonstrate that A3 is essential for VACV viral replication, and that when A3L gene expression is repressed in the absence of tetracyclines, there is no evidence of a productive infection up to 7 DPI.

The function of A6 has been previously studied in VACVs with epitope-tagged A6 and temperature-sensitive mutants [48], as well as IPTG-inducible viruses generated using *lac* operon elements [49, 65]. These studies have shown that A6 is a late gene product packaged into the virion core, is essential for virion membrane synthesis, and plays a role in the localization of virion membranes to viral factories [48, 49, 65]. The *lac* operon-based virus expressing A6L was shown to replicate only in the presence of IPTG, although about 10-fold less efficiently than the parental virus [49]. In our study, viP_11_A6L was able to replicate indistinguishably from wild-type VACV in the presence of DOX both *in vitro* and *in vivo*, suggesting that our genetic modifications did not attenuate the virus.

In future studies, the safety of replication-inducible vectors can be confirmed in immunocompromised or other more rigorous immunodeficient (e.g., SCID) mouse models by evaluating weight loss, clinical signs, mortality, and replication in the presence of DOX. In addition, the immunogenicity of the inducible vectors can be compared to wild-type and parental viruses to make sure that they remain fully immunogenic, and therefore maintain their vaccine and therapeutic efficacy, even under antibiotic treatment. Further modifications to the vectors (i.e., inactivation/deletion of the thymidine kinase and vaccinia growth factor genes) would enhance their use as oncolytic viruses. Finally, marker-free versions of these replication-inducible vectors (i.e., without expression of *gpt* and EGFP) could be developed by transient dominant selection or other methods [66]. In fact, we have recently developed a new method that can produce replication-inducible vectors that are free of screening and selectable markers and express multiple genes of interest, in as little as one week [67].

## Conclusions

Replication-competent VACVs induce strong, long-lived humoral and cell-mediated immune responses and are effective oncolytic viruses [61]. Thus, VACVs that depend on tetracyclines for replication can be used as safer vectors for the development of live recombinant vaccines, oncolytic therapies, and even next-generation smallpox vaccines, provided that future studies confirm immunogenicity is comparable to wild-type virus. Here we show that elements of the *tet* operon can be used to generate VACVs inducibly expressing the A3 or A6 virion proteins that replicate *in vitro* indistinguishably from wild-type VACV in the presence of tetracyclines, but only abortively infect cells in the absence of antibiotics. Similarly, a VACV inducibly expressing A6L replicated in mice at the same level as wild-type VACV in the presence of tetracyclines but was not detected in the absence of antibiotics. These replication-inducible VACVs have the potential to be used for the development of safer, next-generation recombinant live vaccines. Specifically, in the event an individual experiences an adverse reaction after vaccination due to uncontrolled viral replication, the simple cessation of tetracycline treatment should prevent further viral replication and progression of the complication. Recent cases of post-vaccinial encephalitis and progressive vaccinia in military personnel vaccinated in preparation for deployment overseas [68–70] emphasize the benefits of a replication-inducible smallpox vaccine, as the simple withdrawal of tetracycline antibiotic treatment would likely have promoted the clearance of VACV in these individuals. Likewise, cases of adverse reactions due to inadvertent inoculation of VACV from vacinees to household, recreational, or sexual contacts [71–74] would have been altogether prevented in individuals not undergoing tetracycline antibiotic therapy. In addition, our VACV vectors still express heterologous genes (i.e., EGFP) in the absence of tetracyclines, enabling their possible use as a safer, replication-defective vaccine platform. We have recently developed a replication-inducible Zika virus vaccine candidate that expresses Zika virus virus-like particles from VACV in the absence of tetracyclines. Our vectors could also be used for oncolytic therapies, where treatment could be temporally controlled by the administration of tetracyclines (allowing virus replication and oncolytic action) and subsequent withdrawal (stopping virus replication).

## Materials and methods

### Cells and viruses

Cell lines were obtained from the American Type Culture Collection (ATCC, Rockville, MD, USA). African green monkey BS-C-1 (CCL-26) and human HeLa S3 (CCL-2.2) cells were grown in Dulbecco’s modified Eagle medium (D-MEM; Life Technologies, Gaithersburg, MD, USA) supplemented with 10% tetracycline-tested fetal bovine serum (Atlanta Biologicals, Flowery Branch, GA, USA), MEM vitamin solution, 200 mM L-glutamine, and MEM non-essential amino acids (Gibco, Grand Island, NY, USA). All cells were grown at 37°C in 5% CO_2_. The L-variant of VACV strain Western Reserve (WR) was obtained from ATCC (VR-2035) and a clone (9.2.4.8) derived by sequential plaque purification was used to generate the recombinant viruses in this study [56]. High-titer stocks of VACV were obtained in HeLa S3 cells and titered in BS-C-1 cells.

### Animals

Five-week-old female (BALB/cJ × C57BL/6J) normal hybrid (CB6F_1_/J) mice (stock #100007) were purchased from Jackson Laboratory and maintained in accordance with animal care protocols approved by the Institutional Animal Care and Use Committee at the University of Connecticut (Protocol Number A16-029). All inoculations were performed under isoflurane anesthesia. Weight, clinical signs (ruffled fur, hunched posture, decreased activity), and mortality were assessed daily. Euthanasia (weight loss ≥ 25%) was performed by carbon dioxide overdose followed by cervical dislocation.

### Construction of the VACV transfer vectors

The schematic representation of the VACV transfer vector backbone used for the generation of the recombinant VACVs is shown in Fig 1. The transfer vectors were generated in multiple steps by a combination of DNA synthesis (ATUM, Newark, CA, USA), PCR cloning, and standard subcloning, using engineered restriction endonuclease sites (not shown) to facilitate construction. The *gpt*-EGFP fusion gene for combined *gpt* selection and EGFP screening was developed by DNA synthesis of the *E*. *coli gpt* gene (based on the sequence in plasmid pMSG, GenBank:U13860) and the EGFP gene (based on the sequence in plasmid pEGFP-1, GenBank:U55761), using a previously developed strategy [75]. The *tetR* gene (based on GenBank:X00694) was synthesized with an internal VACV early transcriptional termination sequence (TTTTTNT) removed from the middle of the gene (Leu codon at position 358 changed from TTA to CTT) to ensure early gene expression. The tetR and *gpt*-EGFP genes were placed under back-to-back P_E/L_ synthetic promoters (sequence TATTTATATTCCAAAAAAAAAAAATAAAATTTCAATTTTTAACTGCAGTTAAAAATTGAAATTTTATTTTTTTTTTTTGGAATATAAATA) [54]. The transfer vectors also contained the putative E8R or A3L promoter region or a modified P_11_ late VACV promoter with a *tet* operator (O_2_) [25] placed immediately after the late transcriptional initiator element sequences, as shown in Table 1. Each cassette was surrounded by 600 bp of VACV genomic sequences to the left and to the right of the intergenic regions shown in Fig 1 (based on GenBank:NC_006998) to direct homologous recombination and insertion of the cassettes within the appropriate genomic locations. All plasmids were sequenced after synthesis or PCR cloning to confirm sequence identity.

### Generation of VACVs and preparation of high-titer stocks

Recombinant VACVs were generated by standard homologous recombination after transfection of the transfer vectors with FuGENE HD transfection reagent (Promega, Madison, WI, USA) into BS-C-1 cell monolayers infected 2 h previously with VACV WR clone 9.2.4.8 at an MOI of 0.05. Recombinant *gpt*-expressing VACVs were plaque purified from transfection lysates in BS-C-1 cells using selection medium (25 μg/ml mycophenolic acid, 250 μg/ml xanthine, and 15 μg/ml hypoxanthine) [76] in the presence of 1 μg/ml DOX (doxycycline hyclate ≥ 98% TLC, Sigma-Aldrich). EGFP^+^ plaques were visualized under a Carl Zeiss Axio Observer D1 inverted fluorescence microscope (Oberkochen, Germany) using an XF100-2 (EGFP) filter (Omega Optical, Brattleboro, VT, USA). All VACVs were plaque purified at least four times to eliminate contamination with the parental virus. High-titer stocks were generated by infecting HeLa S3 cells with the VACVs at an MOI of 0.1 in the presence of 1 μg/ml DOX. Infected cells were harvested 4 DPI by centrifugation at 300 × *g* for 10 min and resuspension in D-MEM without tetracyclines. Cells were then lysed by freezing and thawing, sonicated, and trypsinized. Finally, cell lysates were clarified to remove contaminating cell debris by a second round of sonication and centrifugation at 500 × *g* for 10 min. For use in inoculation, these stocks were amplified in HeLaS3 cells, processed as described above, then pelleted through a 36% sucrose cushion [77].

### Analysis of recombinant VACV stability and purity

To detect any residual parental VACV after plaque purification, EGFP expression was confirmed by fluorescence microscopy. Briefly, plaque assays were performed on BS-C-1 cell monolayers in 6-well plates in the absence or presence of 1 μg/ml DOX using high-titer stocks. After 2 days of incubation at 37°C, plaques were analyzed by both brightfield and fluorescence microscopy to detect any EGFP-negative plaques that could be present and would represent unstable recombinants or recombinants needing further plaque purification. The genomic organization of each recombinant VACV around the insertion site was checked by PCR analysis of viral DNA purified using a small-scale method employing micrococcal nuclease [78]. The primer sequences used are shown in Table 2 and their relative locations in Figs 1B–1D. The primer combinations used for PCR analysis included 1–3, 2–4, 3–4, 1–6, 2–5, 5–6, 1–8, 2–7, and 7–8 (Fig 1). As a positive control for VACV DNA, primers 9 and 10 were used to amplify a region of the I8R gene.

### The effect of DOX on plaque formation

The ability of the VACVs to replicate in the absence or presence of inducer (DOX) was investigated by standard plaque assay. Briefly, near-confluent BS-C-1 cell monolayers in 24-well plates were infected with the VACVs at approximately 5–20 plaque-forming units (PFU)/well in the absence or presence of 1 μg/ml DOX and incubated at 37°C for 2 or 7 days. Cells were stained and fixed with 0.5% crystal violet in 10% ethanol/20% formaldehyde and isolated viral plaques were imaged with a digital camera or an inverted microscope.

For the analysis of plaque formation by fluorescence microscopy, near-confluent BS-C-1 cell monolayers in 24-well plates were infected with the VACVs at 5–20 PFU/well in the absence or presence of 1 μg/ml DOX. Plaques and infected cells were imaged at 2, 4, 6, 8, 10, and 12 DPI. In a subset of wells infected with the VACVs in the absence of inducer, DOX was added at 2, 4, 6, 8, or 10 DPI and any plaques that formed were imaged 2 days later.

### Effect of DOX on plaque size

The size of the plaques formed by the VACVs in the absence or presence of DOX was investigated by plaque assay. Briefly, near-confluent BS-C-1 cell monolayers in 12-well plates were infected with the VACVs at 30 PFU/well in the absence or presence of 1, 10, 100, or 1000 ng/ml DOX and incubated at 37°C for 36 h. Cells were stained and fixed with 0.5% crystal violet in 10% ethanol/20% formaldehyde and the radius of isolated plaques was measured under an inverted microscope with measurement-capable software (AxioVision 4.8.1, Carl Zeiss).

### Effect of DOX on viral replication *in vitro*

Triplicate monolayers of near-confluent BS-C-1 cells were infected with the VACVs at an MOI of 0.01 in 24-well plates in the absence of tetracyclines. After 1 h, supernatants were aspirated and replaced with medium containing 0, 1, 10, 100, or 1000 ng/ml of DOX. Immediately after this step (to determine input titer) or after 48 h of incubation at 37°C (to determine virus yield), cells were scraped, centrifuged, and resuspended in 0.5 ml of D-MEM. Samples were processed in three cycles of freeze/thaws and sonication, followed by trypsinization, sonication, and centrifugation for 10 min at 500 × *g* to clarify. Virus titers were then determined by a plaque assay on BS-C-1 cells (in duplicate) in the presence of 1 μg/ml DOX.

### Transient complementation

Plasmids expressing the A6L or A3L genes under the VACV P_11_ late promoter (pP_11_A6L, or pP_11_A3L, respectively) were generated by PCR cloning with primers 11–12 (A6L) or 13–14 (A3L) (Table 2). Primers were designed as in [35]. Monolayers of near-confluent BS-C-1 cells were infected with viP_11_A6L or viP_11_A3L at an MOI of 0.01 in the absence of tetracyclines in 24-well plates. After 1 hour, infected monolayers were either collected to determine input titers, or were transfected using FuGENE HD transfection reagent with 0.5 μg plasmid expressing the A6L or A3L genes, under the control of the P_11_ promoter (pP_11_A6L, or pP_11_A3L, respectively), or no plasmid (mock). Cells were incubated for 48 h at 37°C in the absence or presence of 1 μg/ml DOX, then scraped, centrifuged, and resuspended in 0.5 ml of D-MEM. Virus titers were then determined by a plaque assay on BS-C-1 cells (in duplicate) in the presence of 1 μg/ml of DOX.

### viP_11_A6L *in vivo* studies

Groups of five CB6F_1_/J mice were established based on average body weight over three consecutive days. Each group was formed so that mean body weight and standard deviation between groups were approximately equal. At 1 day prior to infection, DOX was filter-sterilized and diluted in autoclaved water at 2, 0.25, 0.125, 0.025, and 0.005 mg/ml, and provided *ad libitum* to the appropriate group [56]. DOX water was freshly diluted and replaced every other day through Day 14. On day 0, all groups were inoculated intranasally (~10 μl per naris) with ~5 × 10^4^ PFU viP_11_A6L in a final volume of 20 μl sterile PBS. Weight, clinical signs and mortality were assessed daily until 14 DPI. Animals were euthanized by CO_2_ overdose and cervical dislocation if weight loss ≥ 25%.

To evaluate weight loss and survival, groups of 10 CB6F_1_/J mice were established, and 0.125 mg/ml DOX in drinking water was provided to appropriate groups as described above. On day 0, groups were inoculated intranasally (~10 μl per naris) with a lethal dose of ~2 × 10^6^ PFU virus (viP_11_A6L or WR) in a final volume of 20 μl sterile PBS. Weight, clinical signs and mortality were assessed daily until 14 DPI. Animals were euthanized by CO_2_ overdose and cervical dislocation if weight loss ≥ 25%.

### Replication of viP_11_A6L in ovaries

To assess viral replication in ovaries, groups of five CB6F_1_/J mice were inoculated intraperitoneally with ~2 × 10^6^ PFU viP_11_A6L or WR, in the presence or absence of DOX. On day 6 post-infection, mice were euthanized and ovaries collected for processing. Ovaries were weighed and homogenized in 10% volume D-MEM by weight. Homogenates were processed in three cycles of freeze/thaws and sonication, followed by trypsinization, sonication, and centrifugation for 10 min at 500 × *g* to clarify. Supernatants were used to titrate virus in 6-well plates of near-confluent BS-C-1 cells in the presence of 1 μg/ml DOX.

### Statistical analyses and image processing

Statistical analyses were performed with GraphPad Prism v. 7.0c (GraphPad Software, La Jolla, CA, USA). Images were processed in Adobe Photoshop CS6 (Adobe Systems, San Jose, CA, USA) with no manipulations other than for contrast.
